# Salvianolic acid B synergizes with azoles against *Candida glabrata* cells in vulvovaginal candidiasis

**DOI:** 10.3389/fcimb.2026.1838295

**Published:** 2026-05-22

**Authors:** Yisheng Chen, Yuping Zhang, Loukaiyi Lu, Qiang Wang, Jing Gao, Chunmei Ying

**Affiliations:** 1 Obstetrics & Gynecology Hospital of Fudan University, Shanghai Key Lab of Reproduction and Development, Shanghai Key Lab of Female Reproductive Endocrine Related Diseases, Shanghai, China; 2 Joint Laboratory for Biomedical Research and Pharmaceutical Innovation, The Unit of Pathogenic Fungal Infection & Host Immunity, Shanghai Institute of Materia Medica, Shanghai Institute of Immunity and Infection, Chinese Academy of Sciences, Shanghai, China

**Keywords:** antifungal activity, azole synergist, *Candida glabrata*, salvianolic acid B, vulvovaginal candidiasis

## Abstract

**Introduction:**

The rising prevalence of antifungal resistance poses a significant threat to global health, with *Candida glabrata* (*C. glabrata*) emerging as a particular concern due to its increasing incidence in clinical settings and the intrinsic low susceptibility to azoles. Salvianolic acid B (SAB), a major bioactive component extracted from *Salvia miltiorrhiza*, exhibits potent antioxidant properties; however, its potential as an antifungal remains unexplored.

**Objectives:**

This study aimed to evaluate the antifungal efficacy of SAB alone and in combination with azoles against *C. glabrata*, both *in vitro* and *in vivo*, and to elucidate the underlying mechanisms.

**Methods:**

The antifungal efficacy of SAB alone and in combination with fluconazole (FLC), itraconazole (ITC), and voriconazole (VRC) was assessed against *C. glabrata*. Synergistic interactions were determined via checkerboard microdilution assays and time-kill studies. The cytotoxicity of SAB was evaluated using lactate dehydrogenase (LDH) release assays, while *in vivo* efficacy was examined using a *Galleria mellonella* (*G. mellonella*) infection model. Additionally, mechanistic insights were obtained through scanning and transmission electron microscopy (SEM/TEM) to visualize morphological and ultrastructural alterations.

**Results:**

SAB exhibited marked synergistic activity with azoles against azole-resistant *C. glabrata* isolates. Importantly, SAB demonstrated negligible cytotoxicity toward mammalian cells. In the *G. mellonella* infection model, combination therapy with FLC and SAB significantly improved larval survival compared to FLC alone. SEM and TEM revealed extensive membrane disruption, cytoplasmic dissolution, and subcellular damage in SAB and FLC treated cells, consistent with the observed synergistic antifungal activity.

**Conclusion:**

SAB synergistically enhances azoles activity against drug-resistant *C. glabrata* both *in vitro* and *in vivo*, with mechanisms involving membrane disruption and ultrastructural damage. These findings position SAB as a promising natural adjuvant for combination therapy against drug-resistant *Candida* infections.

## Introduction

Vulvovaginal candidiasis (VVC) affects millions of women worldwide (San Juan Galán et al., 2023), with approximately 70% experiencing at least one episode during their lifetime (Cornely et al., 2025; Yano et al., 2019). Although *Candida albicans* remains the most commonly fungal pathogen, *Candida glabrata* has emerged as the second most prevalent species in certain geographic regions (Xu et al., 2023). Azoles are the mainstay of VVC treatment; however, reduced susceptibility to these drugs is increasingly observed in VVC patients (Lírio et al., 2019). The management of VVC caused by *C. glabrata* is particularly challenging due to the limited effective antifungal agents and the rising prevalence of drug resistance. Thus, *C. glabrata* is recognized as a high-priority pathogen by the World Health Organization due to its propensity for developing multidrug resistance, and recent epidemiological evidence from China also has highlighted the escalating public health burden of candidemia and antifungal-resistant *Candida* species (Dong et al., 2026; Miao et al., 2026).

Given the slow pace of new antifungal drug development, there is an urgent need for innovative therapeutic strategies, including the use of antifungal synergists to overcome resistance (Campitelli et al., 2017; Liu et al., 2025). Recent studies have demonstrated that alternating antifungal regimens with agents possessing distinct mechanisms of action, such as echinocandins and amphotericin B, can effectively reduce the echinocandin-tolerant subpopulation and minimize the emergence of resistance in *C. glabrata*, providing a compelling rationale for exploring novel combination strategies (Cai et al., 2026). Thus, combination therapy has emerged as a valuable approach to combat antifungal resistance, and natural product based synergists are increasingly being investigated as adjuncts to conventional antifungal agents. Traditional Chinese medicines (TCMs) offer a rich source of candidate compounds for antifungal therapy (Liu and Shao, 2024). TCM-derived monomers, extracts, and powders have demonstrated promising activity against *Candida* species and associated diseases. Numerous natural products, including herbal extracts, synthetic derivatives, and antimicrobial peptides, have shown synergistic potential when combined with conventional antifungals(X. Wang et al., 2025; X. Zhang et al., 2022). The therapeutic advantages of herbal extracts are often attributed to their “multi-component, multi-pathway, and multi-target” mechanisms (Xu et al., 2013).

Salvianolic acid B (SAB) is a major water-soluble phenolic acid extracted from the roots and rhizomes of *Salvia miltiorrhiza* (Danshen), a core herb in TCMs that has been widely used for centuries to promote blood circulation, remove blood stasis, and treat cardiovascular and cerebrovascular diseases (He et al., 2023; Su et al., 2015). SAB is the most abundant hydrophilic bioactive constituent of *Salvia miltiorrhiza* and has been extensively studied for its broad spectrum of pharmacological activities, including antioxidant, anti-inflammatory, anti-apoptotic, anti-fibrotic, anti-tumor, and metabolism-regulating effects across various organs and tissues (He et al., 2023; Ma et al., 2019). The antimicrobial activities of SAB have also been reported in the literature(J. Zhang et al., 2018). One study demonstrated that SAB has antimicrobial effects on methicillin-resistant *Staphylococcus aureus* (MRSA) and *Acinetobacter baumanii* with minimum inhibitory concentrations (MICs) values of 256 and 128 μg/mL, respectively(J. Zhang et al., 2018). More recently, Manish et al. revealed that SAB exhibits *in vitro* activity against various fungal phytopathogens such as *Alternaria* spp. and *Fusarium* spp (Sharma and Manhas, 2022). In addition, synergistic antimicrobial effects have been observed when SAB is combined with levofloxacin or amikacin against MRSA, and with colistin sulfate against *Acinetobacter baumanii*(J. Zhang et al., 2018). SAB has also shown activity against *Colletotrichum* spp., and *Cladosporium herbarum*, with MICs ranging from 31.25 to 100 µg/mL (Sharma and Manhas, 2020). Regarding the role of SAB in different *Candida* species, the ethanol extract of *Salvia miltiorrhiza*, in which SAB is a major hydrophilic bioactive constituent, has been reported to exhibit antifungal effects against *C. albicans* ATCC 18804 (MIC 39 µg/mL), *C. krusei* ATCC 32196 (MIC 39 µg/mL), *C. glabrata* ATCC 2001 (MIC 78 µg/mL), and *C. tropicalis* ATCC 750 (MIC 78 µg/mL) (Lee and Kim, 2016). More recently, SAB was shown to contribute to potent antifungal activity against *C. albicans* when incorporated into a sprayable hydrogel formulation applied to infected wounds, demonstrating its potential utility in combating fungal infections in biomedical settings (Li et al., 2026). Such results highlight the therapeutic potential of SAB and raise the question of whether its synergistic antifungal activity extends to other clinically relevant *Candida* species and drug-resistant fungal pathogens, thereby broadening its potential clinical applications.

Therefore, this study aims to comprehensively evaluate the antifungal efficacy of SAB in combination with azoles against vaginal *C. glabrata* isolates, extending previous work by providing a systematic analysis of synergistic activity, *in vivo* therapeutic efficacy, and ultrastructural mechanisms underlying the observed antifungal effects.

## Materials and methods

### Strains and materials

A total of 30 vaginal *C. glabrata* isolates were obtained from patients diagnosed with VVC at the Obstetrics and Gynecology Hospital of Fudan University. All isolates were identified using MALDI-TOF mass spectrometry (Bruker, Karlsruhe, Germany), stored at -80 °C, and routinely cultured in YPD medium at 30 °C. Fluconazole (FLC), itraconazole (ITC), voriconazole (VRC), and salvianolic acid B (SAB) were purchased from Selleck Chemicals (Shanghai, China) and dissolved in dimethyl sulfoxide (DMSO; Sigma, USA). RPMI 1640 medium and MOPS were obtained from Sigma-Aldrich. HeLa and Caco-2 cell lines were maintained in DMEM supplemented with 10% fetal bovine serum (FBS). *Galleria mellonella* larvae were purchased from Xinanhua Biotechnology Co., Ltd. (China).

### Microdilution checkerboard assay

The synergistic interactions between azoles and SAB were evaluated using a microdilution checkerboard assay in 96-well plates as previously described (Wu et al., 2020). Briefly, twofold serial dilutions of each agent were prepared, with final concentration ranges of 1–64 mg/L for SAB, 0.125–128 mg/L for FLC, and 0.03–32 mg/L for ITC and VRC. Each well was inoculated with a final yeast suspension of approximately 10^4^ CFU/mL. Plates were incubated at 35 °C for 24 h.

MIC was defined as the lowest concentration of each agent that inhibited yeast growth by 50% compared to the drug-free control. The fractional inhibitory concentration index (FICI) was calculated to assess drug interactions using the formula: FICI = (MIC of SAB in combination/MIC of SAB alone) + (MIC of azole in combination/MIC of azole alone) (Gong et al., 2019). Synergy was defined as FICI ≤ 0.5, and addition as 0.5 < FICI ≤ 1.0 (Fernandes et al., 2020).

### Time-kill study

Based on checkerboard assay results, time-dependent killing effects of SAB combined with azoles were assessed in RPMI-1640 medium. Overnight-cultured yeast cells were diluted to an initial inoculum of approximately 10^5^ CFU/mL. Suspensions were treated with SAB alone, azole alone, or their combination at synergistic concentrations in 96-well plates. Untreated cultures served as controls. All plates were incubated with shaking at 30 °C for 24 h. OD_600_ was measured every 15 min using a Biotek Synergy H1 plate reader. Each experiment was performed in triplicate.

### *In vitro* cytotoxicity assay

Cytotoxicity of SAB was evaluated in HeLa and Caco-2 cells using a lactate dehydrogenase (LDH) release assay. Cells (5 × 10^5^ cells/well) were seeded in 96-well plates and cultured in DMEM with 10% FBS at 37 °C in 5% CO_2_. After incubation, the supernatant was removed, and fresh medium containing the test agents was added. Cells were treated with FLC alone (10 and 50 µg/mL), SAB alone (80 and 400 µg/mL), or the combination of FLC + SAB at two dose levels (low: 10 µg/mL FLC + 80 µg/mL SAB; high: 50 µg/mL FLC + 400 µg/mL SAB). Following an additional 24 h incubation, LHD release was measured according to the manufacturer’s instructions. Absorbance was recorded at OD_450_. Cell viability was calculated using the formula: Viability (%) = [(A_450_ of treatment group minus A_450_ of blank control)/(A_450_ of negative control minus A_450_ of blank control)] × 100%. All experiments were conducted in triplicate, and mean values were reported.

### *In vivo* antifungal activity in a *G. mellonella* infection model

The *in vivo* efficacy of SAB combined with FLC was assessed using a *G. mellonella* larval model, as previously described (Bellavita et al., 2022). Larvae weighing 250–330 mg were randomly divided into groups of 20. Overnight cultures of *C. glabrata* (azole-resistant strain CG84 and ATCC 90030) were washed three times with PBS and adjusted to the desired concentration using a Neubauer chamber. The minimum lethal concentration of each strain was first determined over 6 days post-infection. For therapeutic studies, larvae were injected with 10 µL of yeast suspension containing 2 × 10^8^ CFU/mL of *C. glabrata* (ATCC 90030 or CG84) using an insulin syringe. This injection was made into the last left proleg, delivering an inoculum of approximately 2 × 10^6^ CFU per larva. Two hours post-infection, larvae received a second injection of 10 µL containing PBS (control), FLC alone (5 mg/kg for ATCC 90030 or 15 mg/kg for CG84), SAB alone (30 mg/kg for both strains), or the combination of FLC at the respective dose plus SAB (30 mg/kg). Following injections, larvae were placed in Petri dishes and incubated at 37 °C in the dark. Mortality was assessed daily for 6 days; larvae were considered dead when they failed to respond to gentle touch.

### Scanning and transmission electron microscopy

Sample preparation for SEM and TEM was performed as previously described (Zhen et al., 2024). A suspension of ATCC 90030 (1 × 10^5^ CFU/mL) was treated with SAB (8 µg/mL) alone, FLC (1 µg/mL) alone, or their combination at synergistic concentrations at 30 °C for 12 h. Cells were harvested by centrifugation (1×10^7^ cells per sample). For SEM, cells were washed, fixed overnight in glutaraldehyde, washed with sodium cacodylate buffer, and post-fixed in 0.1 M osmium tetroxide for 1 h. Samples were then dehydrated through a graded ethanol series, critical-point dried (K850, Quorum), and sputter-coated with gold (MC1000, Hitachi) for 30 s. Images were captured using a scanning electron microscope (SU8100, Hitachi).

For TEM, cells were fixed overnight in glutaraldehyde, then processed similarly through dehydration and infiltration with acetone-resin mixtures. Samples were embedded in epoxy resin, sectioned using an ultramicrotome (60–80 nm thickness), and stained with 2% uranyl acetate in saturated alcohol and 2.6% lead citrate. PBS-treated cells served as controls. Ultrastructural examination was performed using a transmission electron microscope (HT7800, Hitachi).

### Statistical analysis

All data were analyzed using GraphPad Prism version 10.0 (GraphPad Software, San Diego, CA, USA). Differences among groups were assessed using one-way analysis of variance (ANOVA). Survival curves were generated using the Kaplan-Meier method and compared by the log-rank test. A *p* value < 0.05 was considered significant.

## Results

### *In vitro* synergistic antifungal activity of SAB and azoles against *C. glabrata*

#### Checkerboard combination assay

**Figure 1:**
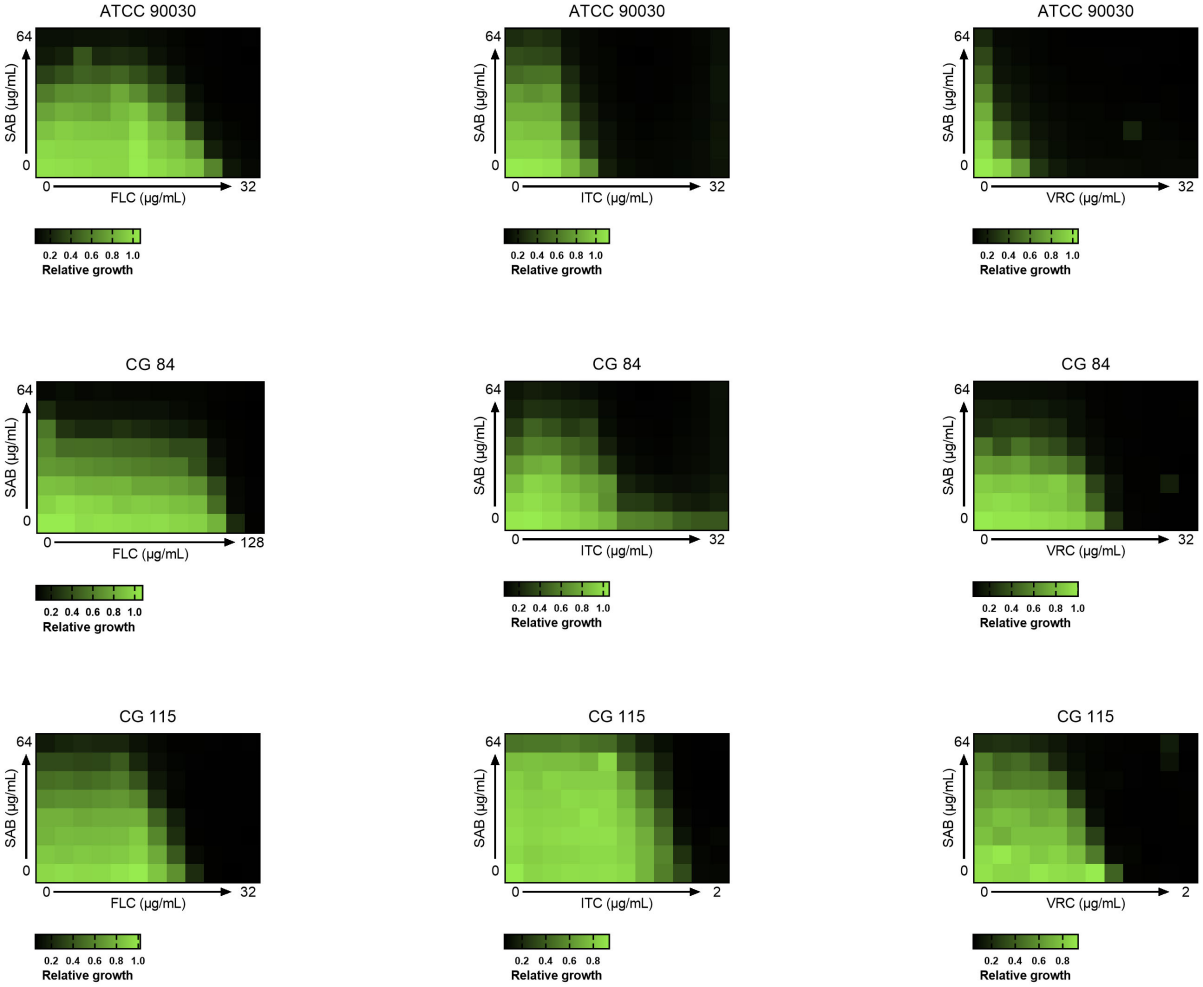
Checkerboard microdilution assays. *C. glabrata* cells were exposed to all the combinations of SAB and azoles. Growth ratios were evaluated after 24 h at 35°C by measuring OD_600_ and normalizing it relative to untreated controls (see gradient bar). Results show the average of three independent experiments. Heatmaps showing the synergistic inhibition. The color gradient represents the percentage of fungal growth relative to the drug-free control.

A total of 31 C*. glabrata* strains (30 clinical isolates and the reference strain ATCC 90030) were evaluated for drug interactions using the checkerboard assay (**Table 1**). Synergistic effects were observed in 29, 20, and 27 clinical isolates for FLC+SAB, ITC+SAB, and VRC+SAB, respectively. Additionally, the ATCC 90030 strain exhibited synergistic effects for all three combinations (FLC+SAB, ITC+SAB, and VRC+SAB). Specifically, in the azole-resistant isolate CG84, the MIC of FLC was reduced 8-fold (from 64 to 8 µg/mL), that of ITC was reduced more than 128-fold (from 64 to 0.5 µg/mL), and that of VRC was reduced 32-fold (from 4 to 0.125 µg/mL) (**Figure 1**). In the azole-sensitive isolate CG115, the MICs of FLC, ITC, and VRC were each reduced 4-fold when combined with SAB, resulting in a FICI of 0.5, indicating synergy (**Figure 1**). For ATCC 90030, the MIC of FLC decreased from 64 to 8 µg/mL and that of SAB decreased from 32 to 8 µg/mL when used in combination, yielding a FICI of 0.375. Additionally, SAB reduced the MICs of ITC (from 4 to 0.5 μg/mL) and VRC (from 1 to 0.06 μg/mL) in this strain (**Figure 1**). Furthermore, no antagonistic interactions were observed for any of the combinations tested.

**Table 1:** Combined drug effects evaluated by the checkerboard assay against *C. glabrata*.

Strain	MIC (μg/mL) alone				MIC (μg/mL) in combination (FICI)		
	FLC	ITC	VRC	SAB	FLC+SAB	ITC+SAB	VRC+SAB
CG16	128	4	2	64	32/2 (**0.281** a)	1/2 (**0.281**)	0.125/4 (**0.125**)
CG19	64	2	1	64	8/8 (**0.25**)	0.25/16 (**0.375**)	0.125/8 (**0.25**)
CG27	128	2	1	64	32/4 (**0.313**)	0.5/16 (**0.5**)	0.125/16 (**0.375**)
CG31	128	>32	4	64	32/4 (**0.313**)	0.5/16 (**0.258**)	0.125/16 (**0.281**)
CG35	>128	>32	8	64	32/16 (**0.375**)	2/2 (**0.063**)	2/4 (**0.313**)
CG80	128	>32	4	32	16/4 (**0.25**)	2/1 (**0.063**)	1/2 (**0.313**)
CG81	>128	>32	4	64	32/16 (**0.375**)	2/2 (**0.063**)	0.5/8 (**0.25**)
CG84	64	>32	4	32	8/8 (**0.375**)	0.5/8 (**0.258**)	0.125/8 (**0.281**)
CG85	64	>32	4	64	8/16 (**0.375**)	0.5/16 (**0.258**)	0.25/16 (**0.313**)
CG113	128	4	2	64	32/16 (**0.5**)	1/1 (**0.266**)	0.125/16 (**0.313**)
CG122	64	4	2	16	16/2 (**0.375**)	1/1 (**0.313**)	0.25/4 (**0.375**)
CG132	64	>32	2	32	16/8 (**0.5**)	1/8 (**0.266**)	0.5/8 (**0.5**)
CG151	128	>32	4	64	32/8 (**0.375**)	2/1 (**0.047**)	1/4 (**0.313**)
CG164	128	>32	4	64	32/2 (**0.281**)	1/2 (**0.047**)	0.5/8 (**0.25**)
CG183	64	4	4	32	16/8 (**0.5**)	1/1 (**0.281**)	0.5/4 (**0.25**)
CG15	4	0.5	0.125	64	1/4 (**0.313**)	0.125/32 (0.75)	0.03/16 (**0.5**)
CG17	32	2	1	64	4/32 (0.625)	0.5/32 (0.75)	0.25/32 (0.75)
CG28	8	1	0.25	32	2/8 (**0.5**)	0.5/4 (0.625)	0.03/8 (**0.375**)
CG79	4	1	0.125	64	1/8 (**0.375**)	0.25/16 (**0.5**)	0.03/16 (**0.5**)
CG83	8	1	0.25	64	2/16 (**0.5**)	0.5/1 (0.516)	0.06/16 (**0.5**)
CG111	16	1	0.25	64	4/4 (**0.313**)	0.25/4 (**0.313**)	0.03/8 (**0.25**)
CG115	4	0.5	0.125	64	1/16 (**0.5**)	0.125/16 (**0.5**)	0.03/16 (**0.5**)
CG120	8	0.5	0.25	64	2/8 (**0.375**)	0.25/16 (0.75)	0.03/8 (**0.25**)
CG135	4	0.25	0.125	64	1/8 (**0.375**)	0.125/16 (0.75)	0.03/16 (**0.5**)
CG150	8	1	0.25	64	2/16 (**0.5**)	0.5/4 (0.563)	0.06/2 (**0.281**)
CG168	16	4	1	64	2/8 (**0.25**)	0.5/8 (**0.25**)	0.125/8 (**0.25**)
CG174	4	0.5	0.125	32	1/8 (**0.5**)	0.125/16 (0.75)	0.03/8 (**0.5**)
CG178	4	0.5	0.25	32	1/4 (**0.375**)	0.25/8 (0.625)	0.03/16 (0.625)
CG191	4	0.5	0.125	32	1/4 (**0.375**)	0.25/8 (0.75)	0.03/16 (0.75)
CG205	32	4	1	64	8/2 (**0.281**)	0.5/8 (**0.25**)	0.06/16 (**0.281**)
ATCC 90030	4	0.5	0.125	32	1/8 (**0.5**)	0.125/4 (**0.375**)	0.03/4 (**0.375**)

MIC, minimum inhibitory concentration; FICI, fractional inhibitory concentration index; FLC, fluconazole; ITC, itraconazole; VRC, voriconazole; SAB, Salvianolic acid B; CG, *C. glabrata*; FICI ≤ 0.5, synergistic; 0.5<FICI ≤ 1, addition. Bold FICI values indicate synergy (FICI ≤ 0.5).

### Time-kill curves

**Figure 2:**
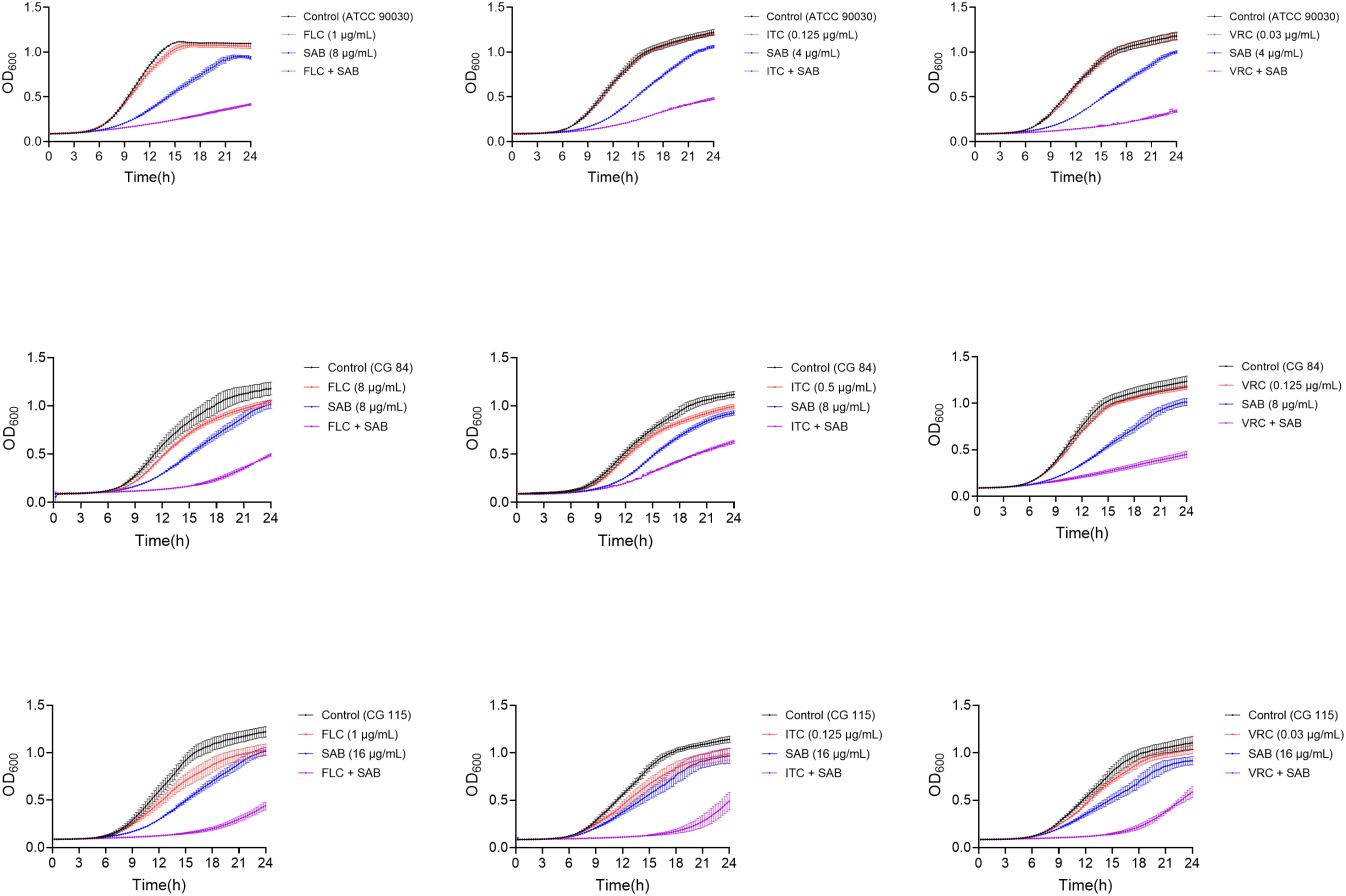
Time-kill curves. *C. glabrata* strains were treated with SAB, FLC, or SAB+FLC, using an initial inoculum of 10^5^ CFU/ml in RPMI-1640 medium. Aliquots were obtained at the indicated time points and detected at OD_600_. The data represent the means ± standard deviations for three independent experiments.

As shown in **Figure 2**, treatment with FLC alone exhibited only a modest suppressive effect on the growth of ATCC 90030, CG84, and CG115, with OD_600_ values rebounding to near-control levels within 24 h. Similarly, SAB monotherapy had a minimal impact on yeast growth over the 24 h observation period. In contrast, the combination of FLC+SAB markedly suppressed yeast growth throughout the 24 h period, maintaining OD_600_ values at the lowest levels among all treated groups.

Likewise, ITC or VRC alone exhibited little impact against yeast growth, whereas their combination with SAB resulted in clearly suppressed growth kinetics. These observations indicate that SAB significantly enhances the antifungal activity of azoles against *C. glabrata* (**Figure 2**).

### Cytotoxicity

**Figure 3:**
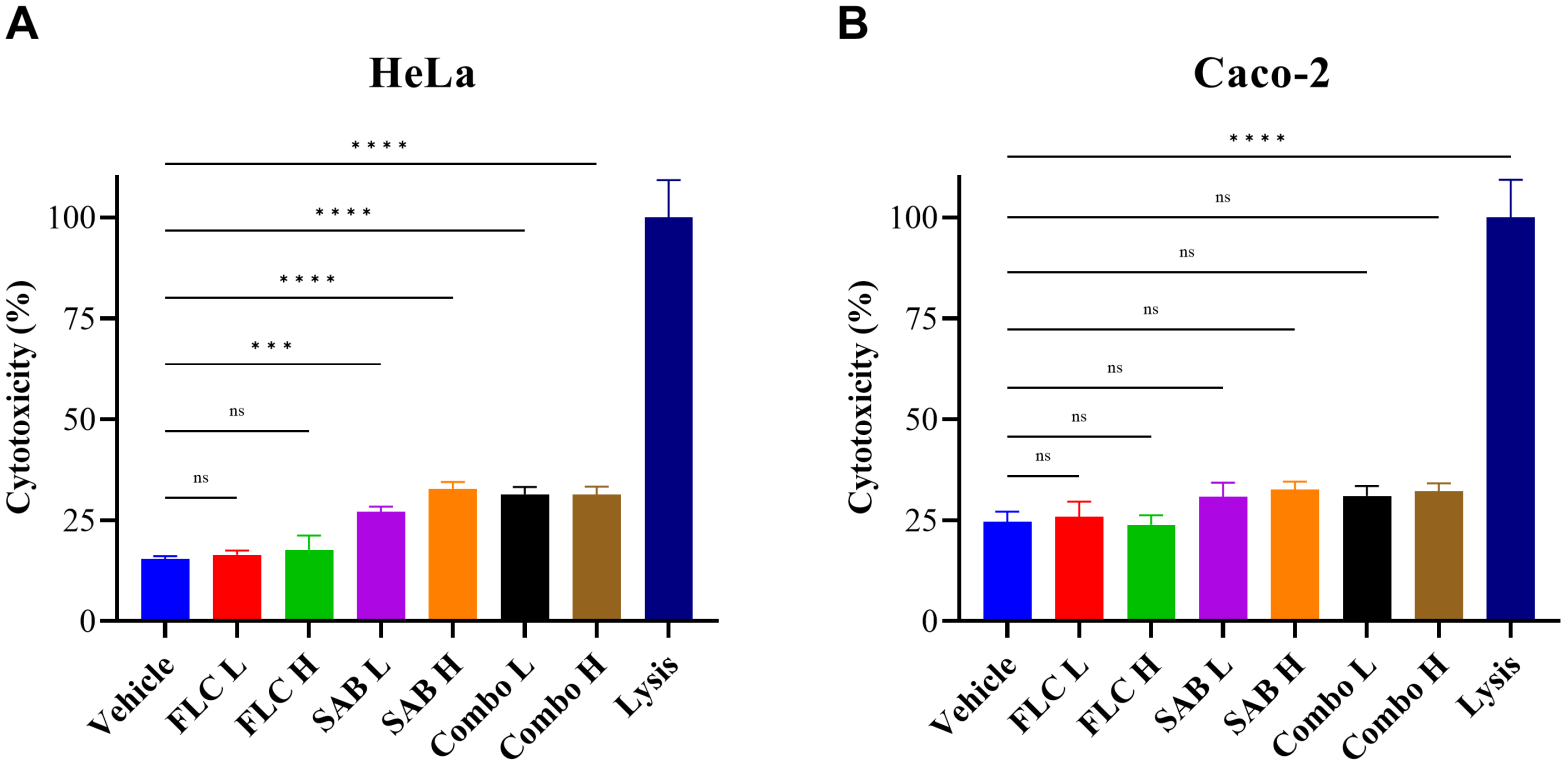
Cytotoxicity of FLC, SAB alone and in combination in HeLa and Caco-2 cells. Cells were treated with FLC alone (10 and 50 µg/mL), SAB alone (80 and 400 µg/mL), FLC+SAB (10 µg/mL FLC + 80 µg/mL SAB; 50 µg/mL FLC + 400 µg/mL SAB) for 24 h. Cytotoxicity was assessed by LDH release assay. **(A)** HeLa cells. **(B)** Caco-2 cells. Data are presented as mean ± SD from five independent experiments. ***p < 0.001, ****p < 0.0001.

The cytotoxicity of SAB alone or in combination with FLC were evaluated in HeLa and Caco-2 cells using an LDH release assay after 24 h of exposure. As shown in **Figure 3A**, compared with the DMSO control, SAB alone or in combination with FLC induced a slight increase in LDH release in HeLa cells. In contrast, no significant increase in LDH release was observed in Caco-2 cells under any of the tested conditions (**Figure 3B**). Specifically, even at the highest combination dose (FLC 50 µg/mL + SAB 400 µg/mL), HeLa cell viability was 68.67 ± 1.93% and Caco−2 viability was 67.88 ± 1.99% (mean ± SD, n=4). Importantly, the LDH release levels remained below 35 % in both cell lines across all concentrations and combination conditions. These results suggest that SAB exhibits negligible cytotoxicity toward host cells at concentrations that effectively inhibit *C. glabrata* growth, supporting its safety profile for potential use in antifungal combination therapy.

### FLC+SAB exhibits *in vivo* synergistic antifungal activity in the *G. mellonella* infection model

The *G. mellonella* larval model has been widely adopted as a cost-effective and ethically accessible invertebrate host for the *in vivo* evaluation of antifungal efficacy (Giammarino et al., 2024). To further evaluate the *in vivo* synergistic efficacy of FLC+SAB, infected larvae were randomly divided into five groups of 20 larvae each, and the experiment was independently repeated twice: (1) PBS-injected (uninfected) control; (2) untreated infected control; (3) infected and treated with FLC alone (5 mg/kg for ATCC 90030, 15 mg/kg for CG84); (4) infected and treated with SAB alone (30 mg/kg); and (5) infected and treated with the FLC+SAB combination.

**Figure 4:**
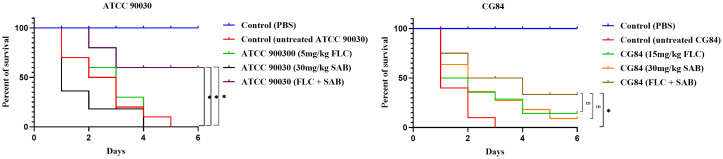
Survival curves for *G. mellonella* infected with *C. glabrata*. Larvae were infected with 2 × 10^8^ CFU/mL of ATCC 90030 or CG84. 10 µL of PBS (control), SAB (30 mg/kg), FLC (5 mg/kg for ATCC 90030; 15 mg/kg for CG84), or FLC+SAB (5 mg/kg FLC + 30 mg/kg SAB for ATCC 90030; 15 mg/kg FLC + 30 mg/kg SAB for CG84) were injected 2 h post-infection. Survival was monitored daily for 6 days with 20 larvae per group, and the experiment was independently repeated twice. The log-rank test was used for statistical analysis.

Over the 6-day observation period, the untreated infected group showed 100% mortality by day 3 for CG84 and by day 5 for ATCC 90030, confirming the virulence of both strains. Treatment with either FLC or SAB alone did not significantly prolong larval survival time. For CG84, FLC alone and SAB alone yielded survival rates of only 15% and 10%, respectively, while for ATCC 90030, both monotherapies resulted in 0% survival by day 5 (**Figure 4**). In contrast, combination therapy with FLC+SAB markedly improved larval survival outcomes. For ATCC 90030, the combination of FLC (5 mg/kg) and SAB (30 mg/kg) significantly delayed larval death and markedly improved survival to 60%, which was significantly greater than that observed with FLC or SAB alone (*p*<0.05, log-rank test). For the azole-resistant strain CG84, FLC (15 mg/kg) or SAB (30 mg/kg) alone delayed larval death but failed to improve survival. However, the combination of FLC (15 mg/kg) and SAB (30 mg/kg) not only delayed larval death but also improved survival to 35%. The uninfected PBS control group maintained 100% survival throughout the experiment, confirming that the observed mortality was attributable to fungal infection rather than injection trauma. These *in vivo* results confirm the synergistic antifungal activity of FLC+SAB against *C. glabrata* in the *G. mellonella* infection model, consistent with the *in vitro* synergistic antifungal activity, and results in significantly enhanced host survival compared to either agent administered alone.

### Cell structure damage observed by SEM and TEM

To directly visualize the impact of drug treatments on fungal cell integrity, we employed SEM and TEM to examine ultrastructural alterations in *C. glabrata* ATCC 90030 cells following exposure to SAB and FLC, alone or in combination.

**Figure 5:**
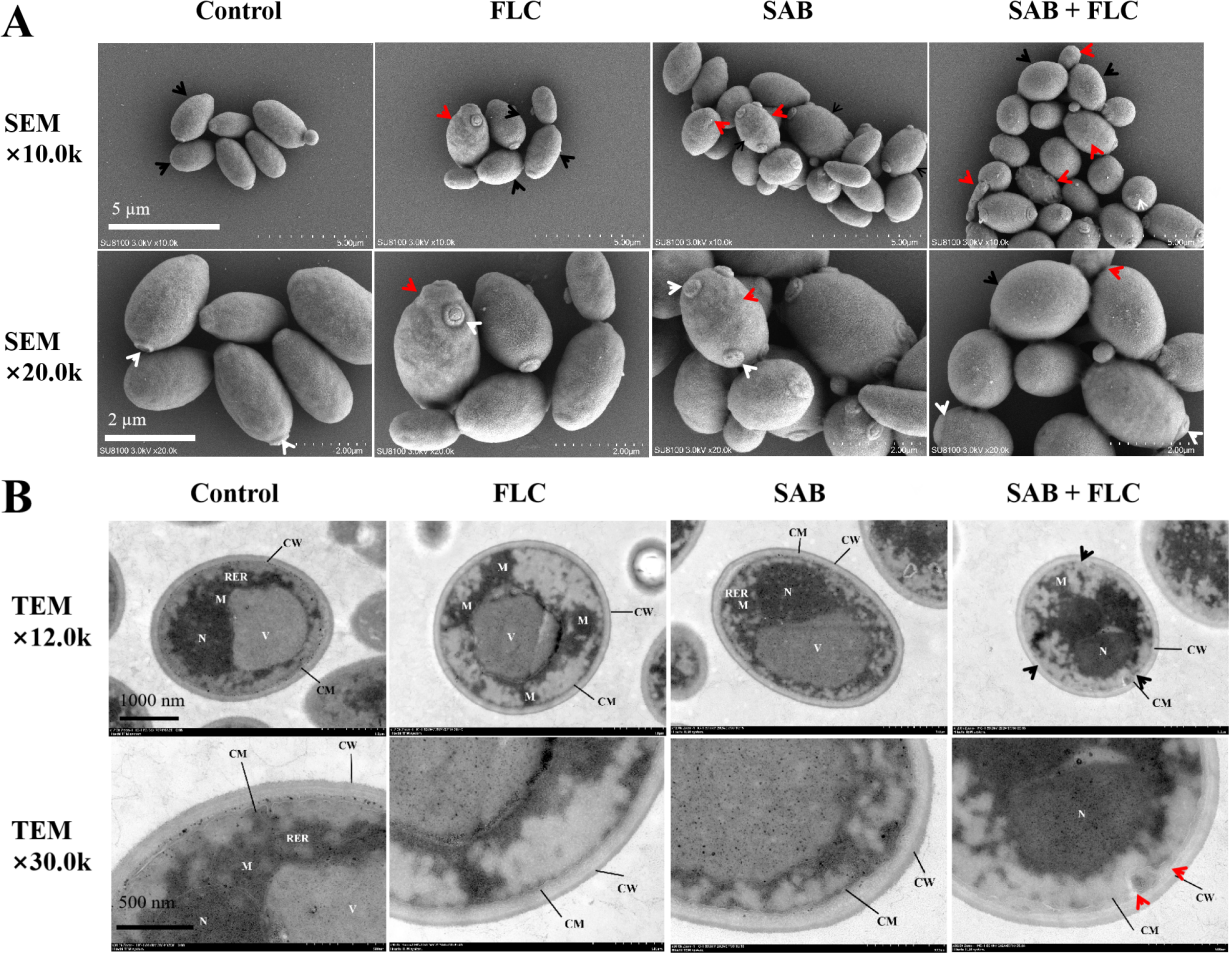
Changes in cell surface and intracellular structures of ATCC 90030 under different drug treatments. Cells were treated with FLC (1 µg/mL) alone, SAB (8 µg/mL) alone, or FLC (1 µg/mL) + SAB (8 µg/mL). **(A)** Scanning electron microscopy (SEM). Scale bar = 2 µm. Black arrow, intact smooth cell surface in control cells; white arrow, bud scar; red arrow, slightly wrinkled cell surface in single-drug treated cells. In the FLC+SAB combination panel, severe surface wrinkling, deep furrows, and cellular collapse are evident. **(B)** Transmission electron microscopy (TEM). Scale bar = 500 nm. CW, cell wall; CM, cell membrane; N, nucleus; M, mitochondrion; V, vacuole. The nucleus (N) is irregular, chromatin condensation, and blurred nuclear membrane; the mitochondria (M) is obviously swollen and larger, and the matrix becomes locally weak. Black arrow, low-electron-density edematous regions in the cytoplasm and dilated/fragmented endoplasmic reticulum, indicative of cytoplasmic dissolution; red arrow, localized shrinkage and collapse of the cell membrane resulting in prominent folds.

SEM examination of untreated control cells revealed a population of yeast cells with characteristically smooth, uniformly rounded surfaces and well-defined budding scars, indicative of intact cell wall architecture. Cells treated with FLC (1 µg/mL) alone exhibited minimal morphological changes: the majority of cells remained largely indistinguishable from controls, displaying regular ovoid shapes and smooth surfaces, with only occasional cells showing slight surface irregularities. Similarly, SAB (8 µg/mL) treatment alone caused subtle alterations, with rare cells manifesting minor surface wrinkling, while the overall population retained normal morphological features. In contrast, cells exposed to the synergistic combination of FLC (1 µg/mL) and SAB (8 µg/mL) displayed pronounced morphological damage. Cells exhibited marked surface wrinkling, deformation, and collapse, with losing their characteristic ovoid shape. The cell surfaces appeared uneven, with deep furrows, prominent folds, and irregular contours. Furthermore, loss of intracellular content suggesting compromise of cell wall integrity (**Figure 5A**).

TEM examination of untreated control cells exhibited typical eukaryotic ultrastructural organization: the cell wall appeared as a uniformly electron-dense outer layer, beneath which the plasma membrane was closely apposed and intact. The cytoplasm was homogeneous, containing a rounded, electron-dense nucleus with dispersed chromatin, normal mitochondria with well-defined cristae, and organized rough endoplasmic reticulum. Treatment with FLC alone induced mild ultrastructural changes, primarily limited to slight irregularities in nuclear shape and subtle mitochondrial swelling in a subset of cells, with the majority of cellular structures remaining intact. SAB alone resulted in comparable mild alterations, with occasional cells showing slightly disorganized cytoplasm and minimal membrane undulation. Conversely, the FLC+SAB combination provoked dramatic subcellular damage. The most conspicuous alterations included pronounced wrinkling and localized collapse of the plasma membrane. The cytoplasm appeared disorganized and exhibited irregular areas of low electron density. Another finding was the presence of chromatin condensation within the nucleus, characterized by dense, marginated aggregates of chromatin material. In addition, mitochondria were markedly swollen and displayed disrupted or absent cristae. Other membranous organelles, including the endoplasmic reticulum, appeared dilated and fragmented. Overall, the combination treatment yielded widespread intracellular architectural destruction (**Figure 5B**). Collectively, these findings provide direct morphological evidence that SAB potentiates the antifungal action of FLC by compromising both membrane integrity and internal cellular organization.

## Discussion

The World Health Organization recently published its first list of fungal priority pathogens, categorizing *Candida albicans* and *Candida auris* as critical priority and listing *Candida glabrata* as a high-priority species (Burki, 2023). In recent years, the incidence of vaginal *C. glabrata* infections has increased significantly (Kan et al., 2023). Although azoles remain the prevently of antifungal therapy, the emergence of drug resistance poses a growing challenge. The limited supply of antifungal medications is a key challenge in preventing fungal resistance (Quindós et al., 2026). Importantly, *C. glabrata* exhibits intrinsically low susceptibility to fluconazole and can rapidly develop drug resistance or cross-resistance (Alikhani et al., 2022). Combination therapy offers the potential to enhance treatment efficacy while reducing adverse effects (Halliday et al., 2026). Evaluating combinations of azoles with synergistic agents therefore represents a promising strategy to overcome azole resistance in *Candida.* Previous work by our group has provided proof-of-concept for this approach using agents with diverse pharmacological mechanisms. Our previous studies demonstrated that miltefosine, an alkylphosphocholine originally developed as an antileishmanial drug, exhibits potent antifungal activity against clinical *Candida* isolates. In *C. krusei*, we showed that miltefosine exerts fungicidal activity by binding to ergosterol in the fungal cell membrane and inducing apoptosis-like cell death characterized by chromatin condensation, and that its combination with amphotericin B resulted in synergistic effects against 25% of planktonic isolates and 18.8% of preformed biofilms, highlighting the potential of membrane-active agents as adjunctive therapy (Wu et al., 2020). Furthermore, we were the first to evaluate the activity of miltefosine against *C. auris*, demonstrating that miltefosine alone showed fungicidal activity and that its combination with amphotericin B exhibited synergy in 3 of 12 isolates tested, with the MIC of amphotericin B being reduced up to 8-fold (Wu et al., 2020).

SAB, the principal bioactive component of *Salvia miltiorrhiza*, exhibits a wide range of pharmacological activities, including antioxidant, anti-/pro-autophagic, anti-/pro-apoptotic, anti-tumor, anti-fibrotic, anti-inflammatory, and metabolism-regulating effects across various organs and tissues (Xiao et al., 2020). However, its antifungal potential has remained largely unexplored. In the present study, we evaluated the effects of combining SAB with three azole antifungals (FLC, ITC, and VRC) against *C. glabrata* clinical isolates. Our findings demonstrate that SAB functions as an effective antifungal synergist, inhibiting *C. glabrata* proliferation. Checkerboard combination assays revealed synergistic or additive activity when FLC, ITC, or VRC was combined with SAB against *C. glabrata* strains. For the azole-resistant *C. glabrata* strains tested, synergistic effects were observed after 24 h of incubation. The degree of synergy appeared to be strain-dependent, likely attributable to phenotypic variations, consistent with previous reports(T. Wang et al., 2021). In line with our results, Khodavaisy et al. found that tacrolimus combined with fluconazole produced synergistic interactions against 60% of *Candida* isolates (Khodavaisy et al., 2023), whereas Gu et al. reported that linezolid combined with FLC exhibited synergy in only 20% of *C. albicans* strains (Gu et al., 2016). In the majority of strains tested in our study, SAB reduced azoles MICs by more than 8-fold, underscoring its potent synergistic potential. Time-kill curves provided further insight into the kinetic effects of the drug combinations on cell viability. The results were consistent with the FICI values, confirming a strong synergistic effect of drug combinations against *C. glabrata*. Moreover, time-kill therapy resulted in growth inhibition without complete eradication, which may help mitigate the emergence of resistance (Joosten et al., 2004). One previous study revealed no adverse effects of SAB at a relatively high dose (2500 μg/mL) (Sharma and Manhas, 2020), our cytotoxicity assays further demonstrated that SAB alone, as well as in combination with FLC, exhibited low cytotoxicity toward both HeLa and Caco-2 cells, indicating that SAB has a favorable safety profile at concentrations that effectively inhibit *C. glabrata* growth, supporting its potential as an adjunctive agent for antifungal combination therapy.

A variety of mini-host models, including *Caenorhabditis elegans*, *Danio rerio*, and *G. mellonella*, have been developed to study fungal pathogenesis. Among those, the *G. mellonella* infection model is cost-effective, easily manipulable, ethically acceptable, and does not require a specific infrastructure (Mroczyńska and Brillowska-Dąbrowska, 2021). Importantly, the ability of *G. mellonella* larvae to survive at near-body ambient temperature (37 °C) makes them particularly suitable for antifungal efficacy studies (Alvarruiz et al., 2025; Jemel et al., 2020). This model has been extensively adopted in medical mycology to assess fungal pathogenicity and the application of antifungal treatments (either alone or in combination with biologically active compounds) (Andrea et al., 2024). Thus, we used the *G. mellonella* infection model to evaluate the *in vivo* synergistic activity of SAB combined with FLC. In this model, larvae were infected with a standardized inoculum of 2 × 10^6^ CFU/larva and treated with strain-specific doses of FLC (5 mg/kg for ATCC 90030; 15 mg/kg for the azole-resistant CG84) alone or in combination with SAB (30 mg/kg). Our results demonstrated that FLC or SAB monotherapy failed to significantly improve larval survival, whereas the FLC + SAB combination significantly enhanced survival to 60% for ATCC 90030 and 35% for CG84, providing effective *in vivo* synergistic antifungal activity of this combination.

Novel antifungal compounds can target multiple cellular processes, including drug efflux, energy metabolism, mitochondrial function, and cell integrity (Stenkiewicz-Witeska and Ene, 2023). Since combination therapies exert multi-target effects, the likelihood of concurrent resistance development to two drugs with distinct mechanisms of action is substantially reduced (Wagner and Ulrich-Merzenich, 2009). Azoles inhibit the fungal cytochrome P450 lanosterol 14alpha-demethylase (CYP51) enzyme and prevent the formation of ergosterol, thereby disrupting the integrity of the fungal cell membrane (Wong et al., 2020). Ultrastructural analysis by SEM and TEM can provide valuable insights into drug interactions. These analyses revealed that cells treated with FLC or SAB alone exhibited minimal morphological changes, whereas the FLC+SAB combination induced cellular damage. SEM analysis demonstrated surface wrinkling, deformation, collapse, and the appearance of deep furrows and irregular contours on the cell surface. TEM provided deeper insight into the subcellular lesions underlying this synergy. The pronounced mitochondrial swelling and loss of cristae suggest that SAB may compromise mitochondrial function, potentially by inducing oxidative stress or disrupting mitochondrial membrane potential, which is consistent with the known pro-oxidant and apoptosis-modulating properties of SAB reported in cancer cells (Xiao et al., 2020). Mitochondrial dysfunction would deplete cellular ATP, thereby impairing the activity of ATP-dependent efflux pumps such as CDR1 and CDR2, which are major determinants of azole resistance in *C. glabrata* (Costa et al., 2016). By inhibiting efflux pump function through energy depletion, SAB may increase intracellular azole accumulation, enhancing target engagement and cell killing. Furthermore, the conspicuous chromatin condensation observed by TEM is a well-established morphological hallmark of apoptosis-like programmed cell death in fungi (Ketelut-Carneiro and Fitzgerald, 2022; Rico-Ramírez et al., 2022), suggesting that SAB, in combination with azoles, may trigger lethal cellular stress responses that culminate in fungal cell death through mechanisms. Collectively, these ultrastructural observations provide direct morphological evidence that SAB potentiates the antifungal activity of azoles by inflicting intracellular damage involving mitochondrial disruption, cytoplasmic dissolution, and chromatin condensation, likely through mechanisms that impair drug efflux and activate programmed cell death pathways. Despite the mechanistic insights provided by our ultrastructural analyses, the precise molecular targets of SAB in *C. glabrata* and the signaling pathways mediating the observed chromatin condensation and mitochondrial damage remain to be fully elucidated. Future studies employing transcriptomic, proteomic, and metabolomic approaches will be essential to dissect the molecular cascades underlying azole+SAB synergy and to identify the specific fungal proteins or pathways targeted by SAB.

In summary, this is the first study to explore the potent combined activity of SAB and azoles against *C. glabrata* in both *in vitro* and *in vivo*. Our encouraging findings, coupled with the low toxicity of SAB, highlight its potential as an adjunctive agent for treating *C. glabrata* infections. Nonetheless, our study have several limitations. First, although our ultrastructural analyses provide morphological evidence for multi-target intracellular damage involving mitochondrial disruption and chromatin condensation, the precise molecular mechanisms underlying the observed synergistic activity remain to be fully elucidated. Second, the study focused exclusively on *C. glabrata*, leaving it unclear whether SAB exerts similar synergistic effects against other clinically relevant fungal pathogens, such as *C. albicans*, *C. tropicalis*, and *C. auris*. Third, the *G. mellonella* model lacks a vaginal tract and the associated vaginal microenvironment, including resident microbiota, acidic pH, and cervicovaginal mucus, that play critical roles in the pathogenesis of VVC and the pharmacokinetics of vaginally administered agents. Moreover, the model does not recapitulate the mucosal immune system, which is central to host defense against vaginal *Candida* infections and may contribute to or modulate the activity of antifungal treatments. Furthermore, the systemic injection used here cannot mimic the localized, topical route of administration typically employed in the clinical management of VVC. Thus, further validation in mammalian models is necessary to better assess the therapeutic potential and translational relevance of this combination.

## Data Availability

The original contributions presented in the study are included in the article/supplementary material. Further inquiries can be directed to the corresponding author.
